# Magnetic susceptibility imaging of human habenula at 3 T

**DOI:** 10.1038/s41598-020-75733-y

**Published:** 2020-11-09

**Authors:** Seulki Yoo, Joo-won Kim, John F. Schenck, Seung-Kyun Lee

**Affiliations:** 1 Center for Neuroscience Imaging Research, Institute for Basic Science (IBS), Suwon, South Korea; 2 Department of Biomedical Engineering, Sungkyunkwan University, Suwon, South Korea; 3 The BioMedical Engineering and Imaging Institute, Icahn School of Medicine at Mount Sinai, New York, NY USA; 4 Department of Radiology, Icahn School of Medicine at Mount Sinai, New York, NY USA; 5 Graduate School of Biomedical Sciences, Icahn School of Medicine at Mount Sinai, New York, NY USA; 6 MRI Laboratory, GE Global Research, Niskayuna, NY USA; 7 Department of Physics, Sungkyunkwan University, Suwon, South Korea; 8 Present Address: Department of Radiology, Baylor College of Medicine, Houston, TX USA

**Keywords:** Brain imaging, Magnetic resonance imaging

## Abstract

The habenula plays an important role in brain reward circuitry and psychiatric conditions. While much work has been done on the function and structure of the habenula in animal models, in vivo imaging studies of the human habenula have been relatively scarce due to its small size, deep brain location, and lack of clear biomarkers for its heterogeneous substructure. In this paper, we report high-resolution (0.5 × 0.5 × 0.8 mm^3^) MRI of the human habenula with quantitative susceptibility mapping (QSM) at 3 T. By analyzing 48 scan datasets collected from 21 healthy subjects, we found that magnetic susceptibility contrast is highly non-uniform within the habenula and across the subjects. In particular, we observed high prevalence of elevated susceptibility in the posterior subregion of the habenula. Correlation analysis between the susceptibility and the effective transverse relaxation rate (R2*) indicated that localized susceptibility enhancement in the habenula is more associated with increased paramagnetic (such as iron) rather than decreased diamagnetic (such as myelin) sources. Our results suggest that high-resolution QSM could make a potentially useful tool for substructure-resolved in vivo habenula imaging, and provide a groundwork for the future development of magnetic susceptibility as a quantitative biomarker for human habenula studies.

## Introduction

The habenular nucleus (Hb) is a bilateral, paired epithalamic structure in the diencephalon that is evolutionarily well-conserved and found in all vertebrates^1,2^. In humans, it appears as two small deep-brain cell masses near the midline on either side of the 3rd ventricle, located adjacent to the pineal gland and protruding from the dorsomedial aspects of the thalamus. It is divided into medial and lateral portions which are distinguished by their anatomical locations as well as functional connections^3^. The habenula is involved in the brain reward circuitry, pain processing, and psychiatric conditions such as major depressive disorder and schizophrenia^4–7^. Since its function plays an important role in various brain systems, the habenula has attracted much interest in recent years in neuroscience as well as in the clinic.

While much study has been conducted on animal models^8^, reports on human in vivo habenula imaging research have been relatively scarce, in part because of its deep-brain location and small size (approximately 30 mm^3^ in volume in both the left and the right hemispheres) compared to the total brain volume. Continued progress in high-resolution MRI has contributed to the growing literature of in vivo human habenula research, including studies of its morphology^9^, connectivity^10^, functional activation^11^, and magnetic susceptibility^12^. In particular, deep brain stimulation of the habenula region assisted by high-resolution MRI has been proposed^13^ and performed^14^ with promising results for drug-resistant major depression.

Anatomical imaging of human habenula has so far primarily relied on relaxation time contrasts stemming from the high density of myelinated neuronal fibers present in the habenula. Myelin leads to reduced T1 and T2, producing hyperintense T1-weighted and hypointense T2-weighted images. This has been exploited in habenula segmentation based on the ratio between T1- and T2-weighted images^15^. In 2015, while investigating neuroanatomical applications of quantitative susceptibility mapping (QSM), Schenck et al. observed noticeable magnetic susceptibility enhancement in the habenula of several (four) volunteers^16^. Similar observations were reported by other groups at 3T^17^ and 7T^18^. Given the success of QSM in generating prominent contrast for several iron-rich regions in the deep brain^19^, these observations raised hope that magnetic susceptibility might similarly provide a novel, conspicuous anatomical contrast for human habenula imaging.

Tissue magnetic properties are sensitively probed by the gradient-recalled-echo (GRE) sequence through its sensitivity to the static-field (B_0_) inhomogeneity in MRI. GRE phase images and effective transverse relaxation time (T2*)-weighted magnitude images derived from the same sequence have long been used to generate anatomical tissue contrast^20–22^. Quantitative maps of the effective transverse relaxation rate, R2* (= 1/T2*) can be derived from echo time (TE)-dependent fitting of T2*-weighted magnitude images. Furthermore, GRE phase images form the basis of susceptibility weighted imaging (SWI) and QSM. Among these, QSM is theoretically capable of higher spatial resolution than other magnetic susceptibility-sensitive methods by mapping the susceptibility source itself rather than its effect on B_0_ disturbances^23^. Being based on phase, QSM retains the relative sign of the tissue susceptibility, providing the distinction between positive (such as iron) and negative (such as myelin) susceptibility sources with respect to water. Such capability contrasts QSM with R2* mapping, which primarily reports on the total amount of B_0_-perturbing susceptibility sources (positive or negative) in the tissue^24^.

In the 2015 report, Schenck et al. used a relatively large (2 mm) slice thickness (voxel size = 0.58 × 0.75 × 2 mm^3^, 0.87 μl) and did not attempt to resolve the substructures of the habenula based on susceptibility. More recently, He et al. conducted a larger study on an older subject group (N = 50, aged 62 ± 8), also at 3 T, and confirmed high conspicuity of habenula in QSM in most of the analyzed subjects (41 out of 44, 93%)^12^. Using higher spatial resolution (voxel size = 0.67 × 0.67 × 1.34 mm^3^, 0.60 μl) the authors showed that the susceptibility enhancement in the habenula was not spatially uniform, but rather occurred primarily in the posterior subregion, which they identified with the lateral habenula. They also suggested the possibility that the source of susceptibility elevation might be low myelin density instead of iron accumulation as was originally proposed by Schenck et al. Importantly, the authors exposed veins of the thalamus as a major confounding factor in susceptibility analysis of the habenula.

In view of the aforementioned prior research, we recognize a few open questions and challenges in habenula susceptibility imaging. First, high spatial resolution, preferably sub-mm in all directions, is necessary to probe the habenula substructure while minimizing partial volume effects and the influence of the veins. Second, the habenula is known to be dense in myelinated fibers but is also believed to be rich in iron^25^. It would be desirable, therefore, to determine which of the two (myelin or iron) dominates observed susceptibility contrast in the habenula. Finally, the positive susceptibility contrast and its posterior localization within the habenula have yet to be confirmed in subjects with a broader age range. Such a study should also ideally compare inter-subject variation with scan-rescan variability which can be significant in small region-of-interest (ROI) measurements.

In this work, we attempted to address these challenges through the following approaches. First, using a high resolution (0.53 × 0.53 × 0.8 mm^3^, 0.22 μl) 3 T imaging protocol, we characterized the magnetic susceptibility distribution within the habenula on 21 volunteers with a wider age range (19–70) than in previous studies. For ROI definition, we borrowed from two published methods^12,26^ to segment the habenula. Consistent with He et al.^12^, we found that veins in and near the habenula interfered with magnetic susceptibility characterization on many subjects. In order to exclude the veins, and to implement consistent processing among all volunteers, we chose to use a single axial slice (called “minimum-vein slice”) that appeared to be the least influenced by the veins for the susceptibility analyses in this work. Second, in an attempt to shed light on the source of localized susceptibility enhancement within the habenula (namely, iron vs low myelin density), we evaluated the correlation between the magnetic susceptibility and the R2* values across the voxels within the habenula. Third, we scanned three volunteers 10 times (each) in different sessions, and investigated the scan-rescan reproducibility of major quantification parameters such as the habenula volume, contrast-to-noise ratio (CNR), and susceptibility-R2* correlation, and compared it with the subject-to-subject variation.

## Results

### Habenula ROI volume and mean susceptibility

For the 21 subjects, the bilaterally averaged habenula volume was 32.2 ± 5.8 mm^3^ (mean ± standard deviation). There was no significant age-volume correlation (Pearson correlation coefficient =  − 0.002, *p* = 0.99; Supplementary Fig. 1A) or gender dependence (unpaired t-test, *p* = 0.08). After selecting the minimum-vein slice, the process of single-slice edge adjustment (see “Methods” for details of ROI definition) resulted in the average increase of the ROI voxel counts by 6.9% and 9.5% for the left and the right habenula, respectively. After adjustment, the mean and standard deviation of the number of voxels in the two-dimensional (2D) habenula ROI bilaterally averaged in the minimum-vein slice were 30.6 and 4.24, respectively, which corresponded to volumes of 6.81 mm^3^ and 0.94 mm^3^. Therefore, approximately one-fifth of the habenula volume was used for susceptibility analysis in our study. For the three volunteers with 10 repeated scans, the average scan-rescan reproducibility in terms of the standard deviation was 6.8% of the mean for the total habenula volume, and 5.6% of the mean for the 2D ROI pixel count.

The mean ± standard deviation of the magnetic susceptibility value in the bilateral habenula, referenced to the cerebrospinal fluid (CSF), was 0.0384 ± 0.0191 ppm for the 21 subjects. There was no significant age-susceptibility correlation (Pearson correlation coefficient = 0.2265, *p* = 0.32; Supplementary Fig. 1B) or gender dependence (unpaired t-test, *p* = 0.12). For the three volunteers with 10 repeated scans, the scan-rescan reproducibility in terms of the standard deviation was 0.0037, 0.0060, and 0.0069 ppm (average = 0.0055 ppm) for volunteers 1, 3, and 4, respectively.

### Visual assessment

The conspicuity of the susceptibility contrast in the habenula was visually assessed and scored by three raters. The scores, averaged over the raters, revealed substantial heterogeneity among the 21 subjects. Specifically, the number of subjects in the score (s) brackets of 1.0 ≤ s < 1.5 (low conspicuity), 1.5 ≤ s < 2.5 (medium), and 2.5 ≤ s ≤ 3.0 (high) were 6, 6, 9, respectively. The mean ± standard deviation of the scores was 2.19 ± 0.77. The intraclass correlation coefficient (ICC) for the three raters was 0.909, indicating a high degree of agreement. The scores did not correlate significantly with the age (Pearson correlation coefficient = 0.1087, *p* = 0.64; Supplementary Fig. 2), or gender (unpaired t-test, *p* = 0.3872). It turned out that the three repeat-scan subjects each fell in one of the three score brackets, with mean scores of 2.17 (volunteer 1), 2.93 (volunteer 3), and 1.30 (volunteer 4). Their scan-rescan variability was relatively low, with standard deviation of 0.28, 0.14, 0.29, respectively. Higher variability tended to be associated with lower conspicuity (volunteer 4).

### Susceptibility-R2* correlation

**Figure 1:**
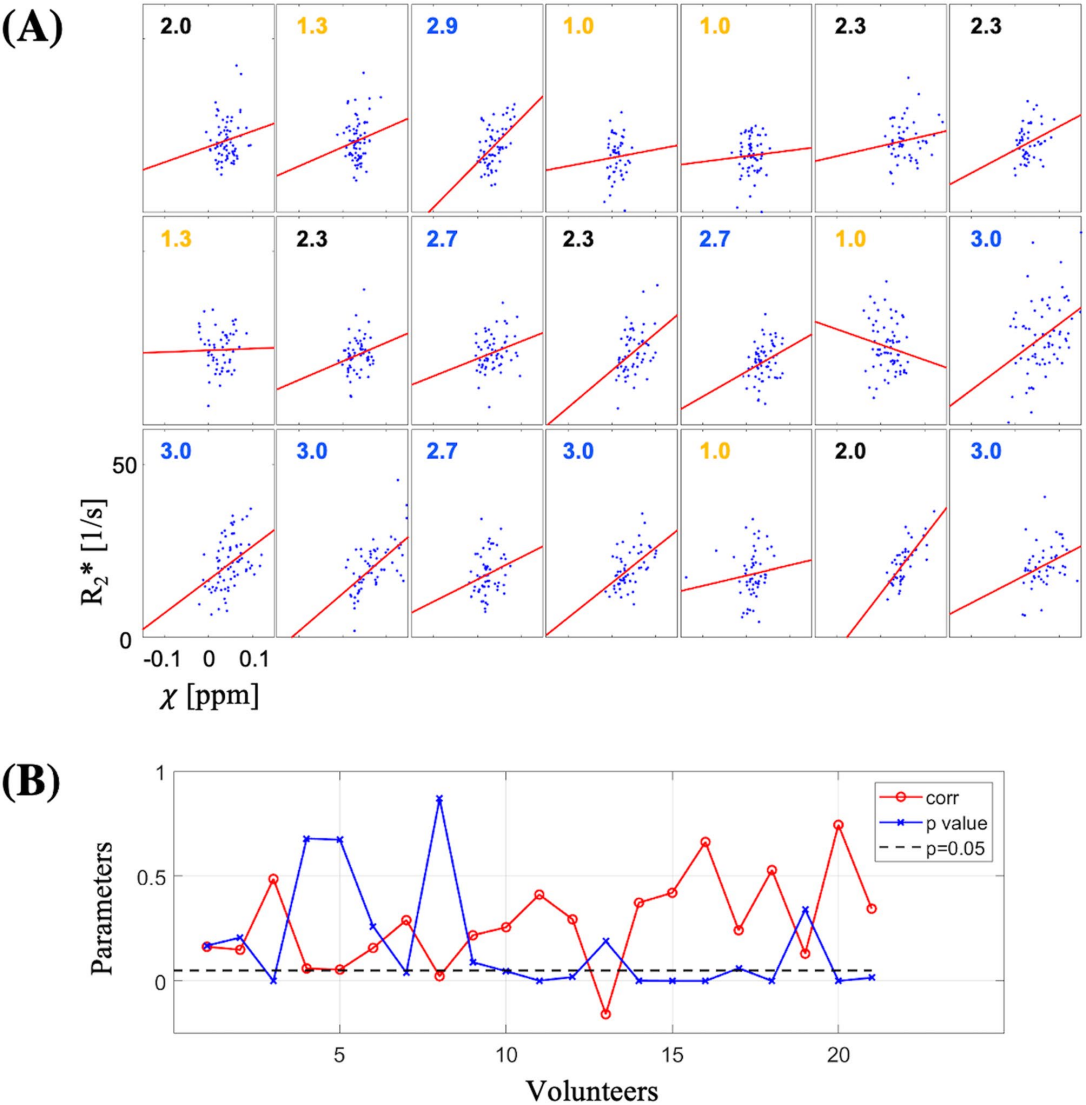
(**A**) R2* vs susceptibility graph for the 21 subjects. The volunteer number increases from the left to right, and then from the top to bottom. On each graph, the visual conspicuity score is indicated in orange (low), black (medium), and blue (high). The solid red lines are linear fits to the data. (**B**) Pearson correlation coefficients (red) between the R2* and susceptibility and the *p* values (blue) for the 21 subjects. Black dashed line indicates *p* = 0.05*.*

Figure 1A shows the plots of R2* vs susceptibility in the bilateral habenula for the 21 subjects along with the linear regression lines. Also indicated on each plot is the subject’s visual conspicuity score which is color-coded according to low (orange), medium (black), and high (blue). All but one subject had a positive regression slope, and the subjects with high conspicuity generally had more distinct positive slopes. The mean and the standard deviation of the slopes were 63.4 [s^−1^/ppm] and 46.7 [s^−1^/ppm], respectively.

Figure 1B shows the Pearson correlation coefficients between the R2* and the susceptibility as well as the associated *p* values for the 21 subjects. The mean ± standard deviation of the correlation coefficient was 0.28 ± 0.22. Eleven of the 21 subjects had a *p* value less than 0.05, indicating a significant correlation. For these subjects, both the mean correlation coefficient (0.44 ± 0.16) and the linear slope (95.8 ± 32.4 [s^−1^/ppm]) were substantially higher than the whole group.

For the three subjects with 10 repeated scans, the scan-rescan standard deviation of the R2*-susceptibility correlation coefficient was 0.14, 0.18, and 0.13 (average = 0.15) for volunteers 1, 3, and 4, respectively (Supplementary Figs. 3, 4).

### Contrast analysis

The standard deviation ${\sigma }_{\chi }$ of the susceptibility in an ROI just outside the habenula (“outer ROI”) served as the measure of noise in the CNR calculation. The value of ${\sigma }_{\chi }$ was relatively uniform across the subjects, with its mean and subject-to-subject variation (standard deviation) given by 0.011 ppm and 0.002 ppm, respectively.

Table 1 shows bilaterally averaged susceptibility contrast values obtained from different regions in and around the habenula for the 21 subjects. The susceptibility difference was the greatest between the peak (5-pixel) region and the rest of the habenula, at 0.0452 ± 0.0147 ppm. This was followed by the susceptibility differences between the posterior and anterior halves of the habenula (0.0258 ± 0.0141 ppm) and between the whole habenula and the outer ROI (0.0142 ± 0.0168 ppm). The difference between the lateral and the medial halves was not statistically significant (paired t-test, *p* = 0.42). When normalized by the outer ROI standard deviation, the CNR values generally followed the same trend, with CNR_peak_ being the highest among the four CNR measures, at 4.40 ± 1.67.

**Table 1:** Bilaterally averaged susceptibility contrasts of the habenula.

Volunteer number	Visual score	$\upchi \left(\mathrm{Hb}\right)-\upchi (\mathrm{outer})$		$\upchi (\mathrm{peak})-\upchi (\mathrm{rest})$		$\upchi (\mathrm{post})-\upchi (\mathrm{ant})$		$\upchi (\mathrm{lat})-\upchi (\mathrm{med})$	
		ppm	CNR_mean_	ppm	CNR_peak_	ppm	CNR_AP_	ppm	CNR_ML_
1	2	− 0.0054	− 0.5970	0.0390	4.4270	0.0177	2.0172	0.0089	1.0268
2	1.3333	0.0061	0.6922	0.0298	2.8971	0.0120	1.0502	− 0.0047	− 0.4432
3	3	0.0248	2.4265	0.0431	4.2255	0.0270	2.6422	− 0.0030	− 0.2941
4	1	0.0042	0.2802	0.0250	2.6418	0.0160	1.6252	− 0.0044	− 0.4613
5	1	0.0104	1.7250	0.0268	3.1327	0.0124	0.9529	− 0.0063	− 1.0417
6	2.3333	0.0308	3.3115	0.0560	5.9889	0.0396	4.2264	0.0068	0.6899
7	2.3333	0.0026	0.3791	0.0422	5.2199	0.0240	2.9950	0.0156	1.8958
8	1.3333	0.0134	1.1419	0.0409	3.5007	0.0285	2.4614	0.0177	1.4945
9	2.3333	0.0281	2.7496	0.0342	3.3439	0.0208	2.0056	− 0.0143	1.4020
10	2.6667	0.0147	1.2500	0.0517	4.3121	0.0386	3.2353	0.0038	− 1.4020
11	2.3333	0.0272	2.1683	0.0411	3.3306	0.0244	2.0340	-0.0117	0.3265
12	2.6667	0.0108	1.3612	0.0388	4.3524	0.0208	2.4817	− 0.0098	− 1.0209
13	1	− 0.0057	− 0.4408	0.0323	2.0910	0.0098	0.5016	− 0.0106	− 1.0580
14	3	0.0298	2.0105	0.0727	5.3526	0.0483	3.6371	− 0.0044	− 0.8327
15	3	0.0037	0.2743	0.0591	4.4791	0.0309	2.3371	0.0171	− 0.1669
16	3	0.0637	7.6586	0.0829	9.7916	0.0595	7.0944	− 0.0160	1.2800
17	2.6667	− 0.0041	− 0.3841	0.0435	4.1440	0.0242	2.3042	0.0153	− 2.1660
18	3	0.0142	1.4935	0.0555	5.8546	0.0376	3.8548	0.0017	1.4562
19	1	− 0.0090	− 0.8513	0.0358	3.3922	− 0.0026	− 0.2382	0.0211	0.1965
20	2	0.0125	0.9157	0.0413	3.7549	0.0174	1.5999	0.0089	0.9814
21	3	0.0251	2.7099	0.0575	6.2198	0.0350	3.7820	0.0120	1.3006
Mean	2.1873	0.0142	1.4417	0.0452	4.4025	0.0258	2.5048	0.0021	0.1789
Stdev	0.7681	0.0168	1.8503	0.0147	1.6659	0.0141	1.5490	0.0116	1.1905

**Figure 2:**
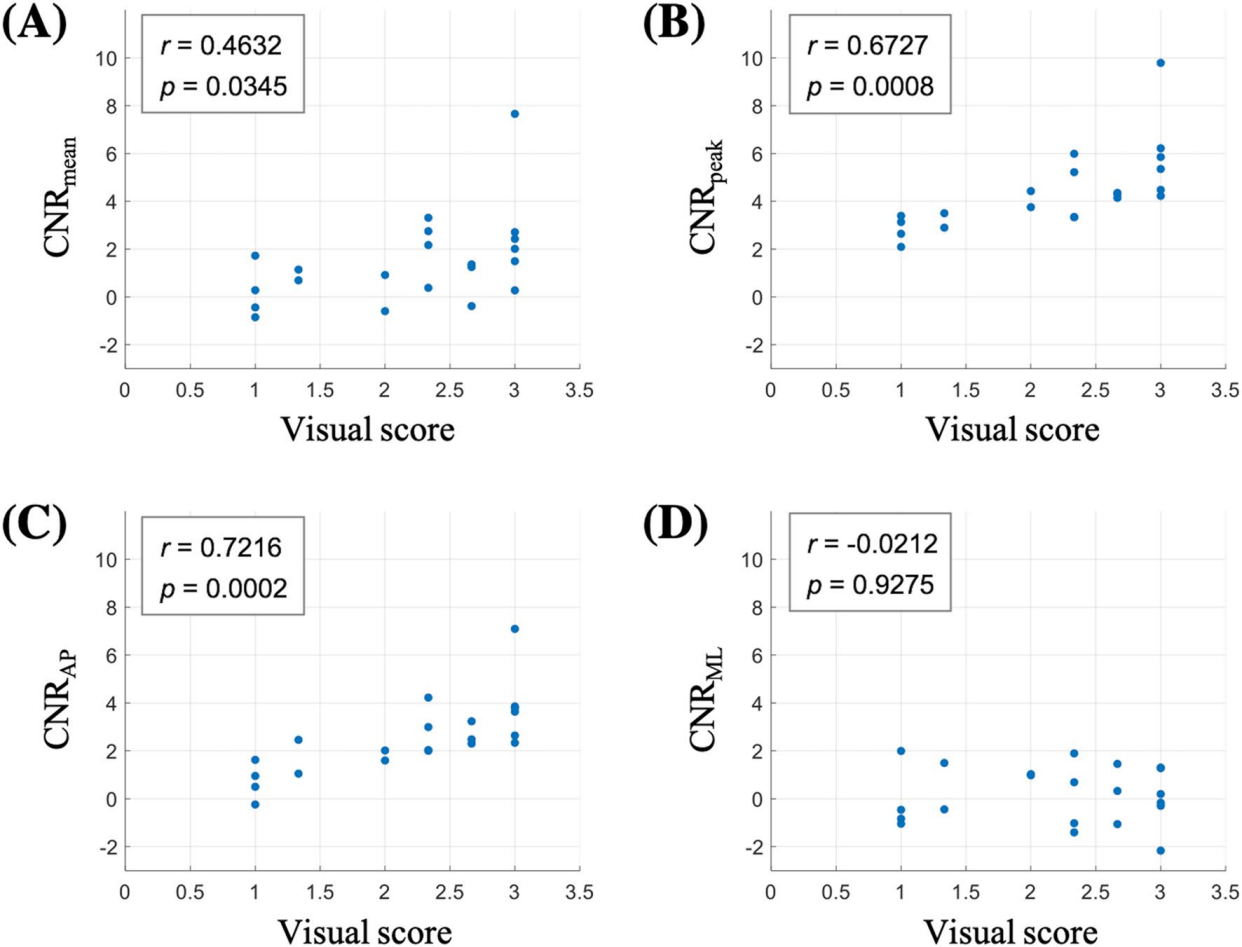
Plots of different susceptibility CNRs vs the visual contrast scores for the 21 subjects. The correlation coefficient (*r*) and the *p* value are indicated on each plot. (**A**) CNR_mean_ was obtained from the difference in the mean susceptibility between the habenula ROI and the outer ROI, defined in “Methods”. (**B**) CNR_peak_ was obtained from the difference between the peak five-voxel cluster in the habenula and the rest of the habenula. (**C**) CNR_AP_ was calculated from the difference between the posterior and anterior subregions within the habenula. (**D**) CNR_ML_ was calculated from the difference between the lateral and medial subregions within the habenula. All CNR values were normalized by the outer-ROI susceptibility standard deviation (Eqs. 1–4).

Interestingly, when we examined the correlation between the CNR and the visual conspicuity scores (Fig. 2), the highest correlation was found for the posterior-anterior CNR (*r* = 0.7216, *p* = 0.0002, Fig. 2C), which was slightly higher than the correlation for the peak-rest CNR (*r* = 0.6727, *p* = 0.0008, Fig. 2B). This suggests that the visual habenula contrast is comparatively well captured by the posterior-anterior susceptibility difference. While the peak-rest CNR was generally higher than CNR_AP_, the former tended to flatten out for low conspicuity subjects, which reduced its correlation with the visual scores. The in-out CNR was more weakly correlated with the scores (*r* = 0.4632, *p* = 0.035, Fig. 2A), while the lateral-medial contrast was not significantly correlated (*r* = − 0.0212, *p* = 0.93, Fig. 2D).

For the repeat-scan volunteers, the average scan-rescan variability (standard deviation over 10 scans) for the four CNR measures ranged from 0.57 to 0.84 (Supplementary Fig. 5), which was less than half of the inter-subject variabilities of 1.19–1.85 (Table 1). In terms of the susceptibility difference itself, the scan-rescan variability was 0.0041–0.0075 ppm, also much smaller than the inter-subject variability of 0.0116–0.0168 ppm (Table 1). The lowest scan-rescan variability in the susceptibility contrast (0.0041 ppm) was observed in the posterior-anterior difference. This is comparable to the theoretical tissue susceptibility uncertainty due to the nuclear spins^27^.

## Discussion

Using high-resolution QSM (with voxel volumes 3–4 times smaller than in previous 3 T studies) and detailed ROI analysis, we have investigated the magnetic susceptibility contrast and its spatial distribution in the habenula of 21 healthy subjects. We found that the majority of the tested subjects (15 out of 21, 71%) showed moderate-to-high, positive susceptibility contrast in the habenula. For these subjects, the susceptibility enhancement was observed predominantly in the posterior subregion of the habenula, in agreement with the previous report^12^.

One of the original motivations of the study was to evaluate QSM as a means to segment out the habenula volume based on its magnetic susceptibility. Previously, QSM was successfully used for the segmentation of deep brain structures such as substantia nigra and red nucleus^28^. In the case of the habenula, however, it was clear from the early data that the susceptibility contrast was highly variable across the subjects, making reliable segmentation based solely on susceptibility difficult. In the method proposed by Lawson et al.^26^, the bilateral habenula was segmented on coronally reformatted T1-weighted MPRAGE images. More recently, He et al.^12^ traced the habenula on short echo-time GRE magnitude images on the axial plane. While the axial view was beneficial in defining the posterior edge of the habenula trigone, the GRE-based method was less effective in defining the lateral boundaries of the habenula against the thalamus compared to the MPRAGE-based method. Given this consideration, in this study we combined coronal-plane MPRAGE segmentation by Lawson et al. with axial-plane manual adjustment.

One disadvantage of such a hybrid approach is the need for an extra registration step between images with different contrasts. Multi-contrast segmentation is not uncommon; for example, myelin content-based semi-automatic habenula segmentation^15^ relied on the ratio between T1-weighted and T2-weighted images registered together. Multi-contrast methods can provide benefits that outweigh risks of misregistration, especially in high resolutions and in the absence of a definitive single contrast. In a previous pilot study, we have investigated T1-weighted-only and GRE-only single-contrast segmentation for habenula susceptibility characterization^29^. We found that the GRE-based method captured the statistically significant R2*-susceptibility correlation as reported here, but the T1-based (Lawson’s) method did not, partly because of missed voxels near the posterior edge. In agreement with He et al.^12^, this suggests that GRE-only segmentation could potentially be an alternative to the hybrid approach for susceptibility imaging. In the present study, however, we decided to use the Lawson’s method at the initial stage of segmentation because of its wider use in habenula imaging.

A related question is whether higher-resolution T1-weighted images with the same voxel size (0.53 × 0.53 × 0.8 mm^3^) as the GRE images would have improved segmentation. We note that regardless of resolution, image registration is still required (as in Kim et al.^15^) because of motion. While matched resolution would generally help, decreased voxel size could prolong the scan and increase motion potential. In order to explore the effect of resolution, we acquired matched-resolution T1-weighted images on one of the subjects (volunteer 3) who had volunteered for the reproducibility runs. The results of the analysis using ROIs segmented with higher-resolution images were consistent with the main results of the present study. Specifically, the mean susceptibility of the habenula (0.0449 ppm), the volume of the minimum-vein slice ROI (7.01 mm^3^), the susceptibility standard deviation in the outer ROI (0.0079 ppm), and the Pearson correlation coefficient between R2* and susceptibility (0.65) were all within the range of the 10-scan reproducibility data. In addition, the descending order of the four CNR values (CNR_peak_, CNR_AP_, CNR_mean_, CNR_ML_) was reproduced. From this, we believe that T1-weighted imaging at higher resolutions would not have changed the main conclusions of the present study.

We have chosen an ROI just outside the habenula in the thalamus (“outer ROI”) to define susceptibility noise in all CNR calculations. This ROI is in a relatively homogenous region with little venous interference and appears well-suited for susceptibility noise calculation. Another possible choice is the CSF in the 3rd ventricle, as used for susceptibility reference (“Segmentation and adjustment of ROI”). A preliminary survey comparing the different noise ROIs showed that our main conclusions regarding CNR did not change with the choice of CSF. For example, CNR_AP_ showed the largest correlation with the visual score with the smallest *p* value (see Supplementary Fig. 6 for details). The mean ± standard deviation (across the subjects) of the susceptibility noise in different ROIs was also comparable: 0.0103 ± 0.0025 ppm (left outer ROI), 0.0110 ± 0.0024 ppm (right outer ROI) and 0.0136 ± 0.0025 ppm (CSF region).

To understand the source of the susceptibility enhancement, we analyzed the correlation between the susceptibility and the R2*. We found that the correlation was positive whenever it was statistically significant. As noted in Zhang et al.^24^, iron and myelin make distinct contributions to susceptibility and R2*. Their involvement can be modeled as^30^ $$\Delta \chi ={ac}_{Fe}-b{c}_{m}$$ and $$\Delta {R}_{2}^{*}=f{c}_{Fe}+g{c}_{m}$$, where susceptibility and R2* changes are expressed in terms of the concentrations of iron ($${c}_{Fe})$$ and myelin ($${c}_{m}$$) with positive coefficients $$a, b, f, g$$. It follows that variation of iron drives both $$\Delta \chi$$ and $$\Delta {R}_{2}^{*}$$ in the same direction, leading to positive correlation, whereas variation of myelin causes opposite changes in susceptibility and R2* leading to negative correlation. This effect has been used in Zhang et al.^24^ to study myelin changes in multiple sclerosis patients. Our observation therefore suggests that the localized susceptibility enhancement in the habenula is more likely dominated by excess iron than myelin deficit, although both may be present in a voxel. Additional parametric mapping to independently measure the myelin content^31^ will further help elucidate this point. It should be noted that in the above model we ignored other sources of susceptibility changes such as calcium or chemically induced frequency shifts. In this work we focused on comparing two primary sources of susceptibility that have been cited in the previous habenula studies^12,16^. We also note that accidental inclusion of the veins in the ROI would have strongly biased the susceptibility-R2* correlation to the positive side. With our conservative choice of the ROI to avoid the veins, and given the high prevalence of statistically significant positive correlations (as opposed to a small number of outliers), we do not believe that our result was substantially biased by the veins.

We note that the average linear slope of 95.8 [s^−1^/ppm] for the subjects with significant correlation is comparable to that observed in the substantia nigra of a patient undergoing iron chelating therapy^32^, 89 [s^−1^/ppm], measured at 3 T with voxel size (0.6 × 0.6 × 1.0 mm^3^) comparable to ours. Assuming that the iron chelation study effectively traced iron-dependent changes in R2* and susceptibility, our measured slope in the habenula appears to be compatible with variations due to iron in the tissue.

In our study, we found that a significant portion (6 out of 21, 29%) of the subjects showed little or no visual susceptibility contrast. This indicates that unlike previously envisioned^16^, magnetic susceptibility alone would not be sufficient for a definitive, universal contrast for visualization of the human habenula. Instead, it could potentially supplement other contrasts such as relaxation and diffusion contrasts, to help delineate different subregions of the habenula in a subset of the population. As to the substructure specificity, we find it intriguing that for all the subjects with visible susceptibility contrast (s > 1), the posterior-side susceptibility was higher than the anterior. This agrees with the previously reported observation^12^. The posterior-interior difference was robust upon repeated scans (scan-rescan variability of 0.0041 ppm), and was the most strongly correlated with the visual score among the four CNR measures analyzed. The underlying source of the observed spatial patterns of the habenula susceptibility is unclear at the moment. While He et al. identified the hyperintense voxels in the habenula with the lateral habenula (in the context of deep brain stimulation)^12^, in our opinion such association would require further investigation. In a 2014 report^33^ Strotmann et al. have used 1 mm isotropic-resolution diffusion tractography to demonstrate distinct fiber orientations for the lateral and the medial habenula. In an ex vivo study^34^, high-resolution (0.06–0.3 mm isotropic) T1 and T2* maps have revealed further differentiation of the lateral habenula into lateral and medial subcomponents consistent with histological results. To our knowledge, systematic in vivo comparison between fiber orientation, myelin density (or its relaxation time surrogates), and QSM in the same subjects is currently lacking. Such study will be instrumental to uncover the source of the observed susceptibility localization in the habenula.

We have repeated the entire procedure of image acquisition and data processing 10 times for three of the 21 volunteers, each representing one of the three (low, medium, high) visual contrast groups. Sources of variability upon repeated scans include scanner drift, random noise, physiological motion, and variations in postprocessing such as ROI segmentation. We found that the scan-rescan variability (standard deviation) of the habenula volume (6.8% of the mean), visual score (24% of the score interval), susceptibility-R2* correlation coefficient (0.15), and the CNR values (0.57–0.84) were low compared to the respective subject-to-subject variations. Our data on scan-rescan variability can provide guidance for the minimum detectable changes in future susceptibility studies of the human habenula. We note that, although parameters such as brain susceptibility, R2*, etc., are normally assumed not to vary significantly over daily, weekly or monthly time scales, the possibility of actual variation has not been exhaustively studied in the literature. It is conceivable, therefore, that processes such as axonal transport of iron or ferritinophagy^35^ might lead to measurable susceptibility changes in the habenula, which merits further study.

We have used a publicly available QSM reconstruction package STI Suite for susceptibility calculation. This package incorporates a dipolar inversion algorithm (STAR-QSM) that suppresses streaking artifacts, and has been ranked high in root-mean-square error in 2016 QSM reconstruction challenge^36^. Unlike R2* calculation, in QSM there is not yet a single gold-standard reconstruction method, and different methods that balance different aspects of reconstruction (such as speed, artifacts, and robustness to anomalies) can produce noticeably different susceptibility maps^36^. We acknowledge that some of the quantitative values reported here, such as the mean susceptibility of the habenula, may change on different algorithms. However, we believe that the following qualitative conclusions of the present study will stand. They are: (i) frequent, but not invariable, observation of enhanced susceptibility in the habenula of healthy adults, (ii) localization of enhanced susceptibility in the posterior habenula, and (iii) tendency of positive correlation between R2* and susceptibility in the habenula. This is because the first two observations were also made in He et al.^12^ where QSM was reconstructed by a different method (truncated k-space division). The third conclusion is backed by the fact that R2* is also often found to enhance in the posterior region of the habenula for high-conspicuity subjects, as shown in Fig. 7. Such observation suggests positive R2*-susceptibility correlation may be a general feature insensitive to reconstruction method.

Image distortion and systematic bias can adversely affect quantitative imaging. In particular, pixel shift and signal loss due to susceptibility-induced B0 inhomogeneity^37,38^ can affect the ROI volume and R2* measurements, respectively. We do not think, however, that B0 inhomogeneity was a significant issue in our study. Unlike the prefrontal and temporal regions, the thalamic region is known to have a relatively uniform B0 field^39^. From our data, we have surveyed the extent to which B0 inhomogeneity could have affected our data. We found that the B0 distribution in the rectangular region shown in Fig. 5 was approximately Gaussian, with a typical two standard deviation of 2 ${\sigma }_{B0}$ = 32 Hz = 0.26 ppm. This is about 1/8 of the pixel bandwidth (240 Hz) in the multi-echo GRE sequence, meaning that about 95% of the voxels in the region experienced less than 1/8 of a pixel shift. The extent was even smaller when we limited the analysis to the habenula only. Likewise, we also found that the local B0 gradient within the habenula, which can affect R2*, was small, being on the order of 1.6 Hz/cm = 0.013 ppm/cm. Using the analytical expression for gradient echo signal^39^, we found that this can lead to R2* overestimation by less than 3%. This is smaller than a typical R2* fitting uncertainty. Therefore, we have not applied additional algorithms for B0-related distortion correction or R2* compensation.

Our study had several limitations. First, the number of volunteers was relatively small. While we collected 48 scan datasets, only 21 were from unique subjects. Our study was designed to include sufficient repeated scans to assess the scan-rescan reliability of the susceptibility measurement in the habenula, in recognition of the potential challenge of imaging a small structure. Second, only a single axial slice from each dataset was analyzed. This precluded analysis of the spatial distribution in the inferior-superior direction and left out many voxels from correlation and contrast analyses. This choice of limited ROI was made to minimize the influence of the veins (which could be detrimental to susceptibility-R2* correlation), while still allowing assessment of the susceptibility distribution in the axial plane. We note that even if a vein does not directly enter the habenula ROI, it can still cause streaking artifacts due to the non-local nature of QSM reconstruction^40^. Despite limitations, we were able to define four distinct CNR parameters from the axial slice, and conduct visual evaluation on the same. While we found that the anterior–posterior contrast was the best correlated with the visual scores, it is possible that other CNR measures on different planes or from volumetric analysis could yield higher correlation. Finally, the multi-echo GRE scan for QSM took a relatively long time (18 min 47 s), making the scan prone to subject motion. In the future, the scan time could be reduced by decreasing the FOV in the slice direction (from the current 96 mm)^41^ and employing higher acceleration factors^42^. Previous research^41^ has shown that reduced FOV coverage of approximately 70 mm in the inferior-superior direction was sufficient to produce susceptibility maps in the deep brain nuclei with < 5% error. Shorter scans will make the study more conducive to the inclusion of other sequences for multi-parametric mapping (e.g. T2, diffusion), provided the signal-to-noise ratio is sufficient.

## Conclusion

In conclusion, we have investigated the magnetic susceptibility contrast and distribution in the human habenula using high-resolution 3 T MRI. We found that susceptibility enhancement in the habenula was non-uniform across the subjects and within the habenula. The susceptibility enhancement was generally positively correlated with R2*, suggesting the role of a paramagnetic source. Our results provide a groundwork for future development and utilization of magnetic susceptibility as a quantitative biomarker for high-resolution imaging of the human habenula.

## Methods

### Participants and data acquisition

Institutional Review Board (IRB) of Sungkyunkwan University approved our study. Our study was performed in full accordance with the local IRB guidelines. Informed consent was obtained from all subjects. Twenty-one healthy subjects (11 males, 10 females, age mean ± standard deviation = 38.81 ± 16.15) were recruited and scanned in a 3 T scanner (Magnetom Prisma, Siemens Healthineers, Erlangen, Germany) with a vendor-provided 20-channel head-and-neck RF receiver array. After a 3-plane localizer, each subject was scanned with a T1-weighted, magnetization-prepared rapid acquisition with gradient echo (MPRAGE) sequence and a 3D multi-echo gradient echo (GRE) sequence for structural and susceptibility-sensitive imaging, respectively. The scans were performed on the axial planes parallel to the anterior commissure-posterior commissure line. MPRAGE covered the whole brain, while slices for the multi-echo GRE scan covered roughly half the brain centered around the posterior commissure. The scan parameters and settings are listed in Table 2. Three of the subjects (1 male and 2 females; volunteers 1, 3, 4) were scanned 10 times in different scan sessions (spanning 2 weeks to 11 months) for scan-rescan reproducibility test.

**Table 2:** Scan parameters and settings.

	T_1_-MPRAGE	Multi-echo gradient echo
TR	2000 ms	47 ms
TE	2.37 ms	7 to 42 in 5 ms (N_echo_ = 8)
Scan duration	3 min 44 s	18 min 47 s
Field of view	230(AP) × 195(LR) × 230(SI)	270(AP) × 186(LR) × 96(SI)
Matrix size	288 × 244 × 288	512 × 352 × 120
Voxel size	0.8 × 0.8 × 0.8 mm^3^	0.53 × 0.53 × 0.8 mm^3^
Flip angle	9°	20°
Pixel bandwidth	250 Hz	240 Hz
Parallel imaging factor	2	2
Acquisition dimension	3D	3D
Scan plane	Axial	Axial
Flow compensation	N/A	First echo, 3 directions

### Data analysis

**Figure 3:**
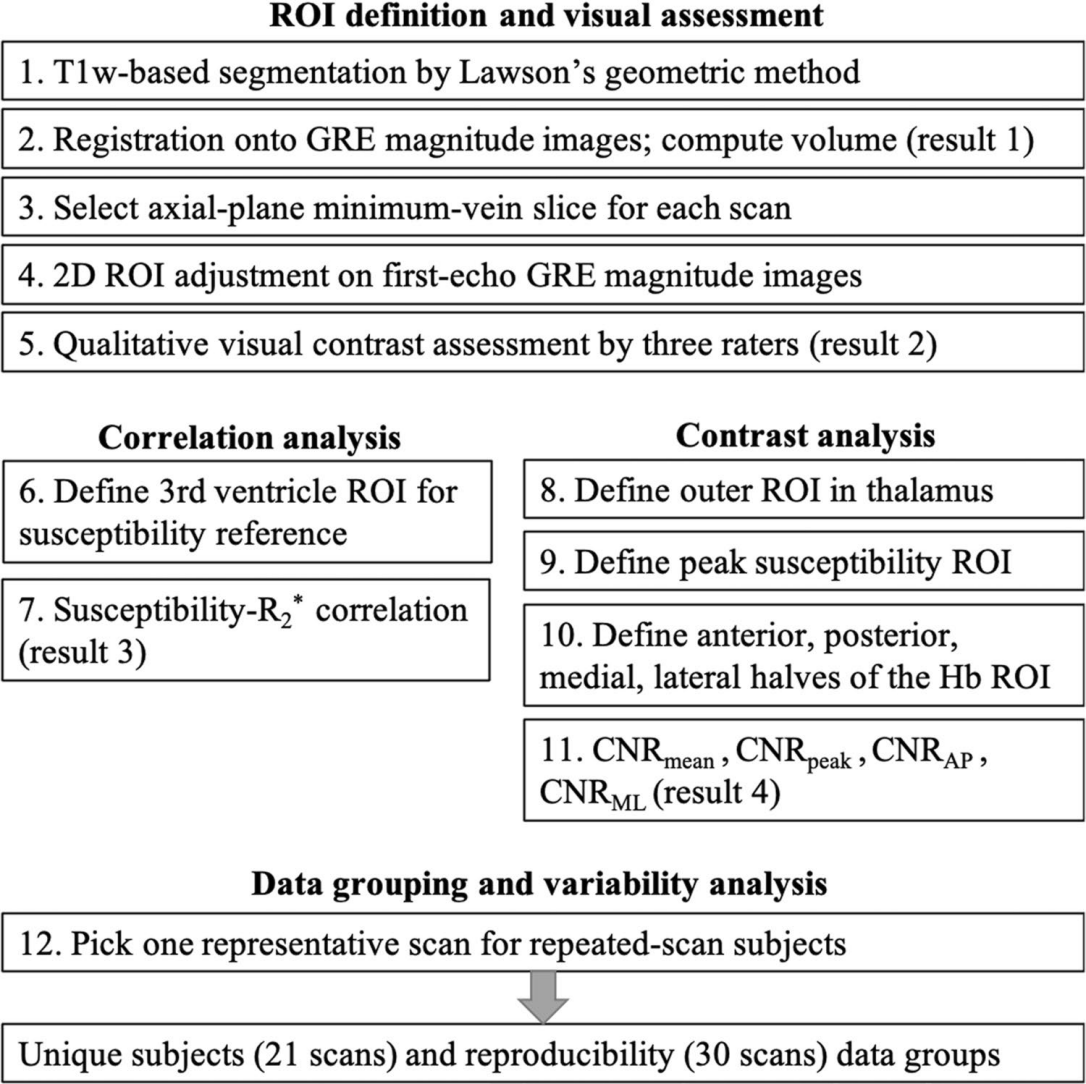
Data processing workflow for habenula susceptibility analysis.

Preprocessing was performed with FSL^43,44^ and Human Connectome Project (HCP) pipeline^45^. T1-weighted images and multi-echo GRE images were registered with Advanced Normalization Tools (ANTs v.2.2.0, https://stnava.github.io/ANTs/). All other image processings including QSM reconstruction and region-of-interest (ROI) analyses were performed in the MATLAB environment (MathWorks, Natick, MA, USA). For statistical processing, we employed the SPSS software (IBM-SPSS, Chicago, IL, USA). The overall data processing workflow in this study is shown in Fig. 3.

#### Image reconstruction

**Figure 4:**
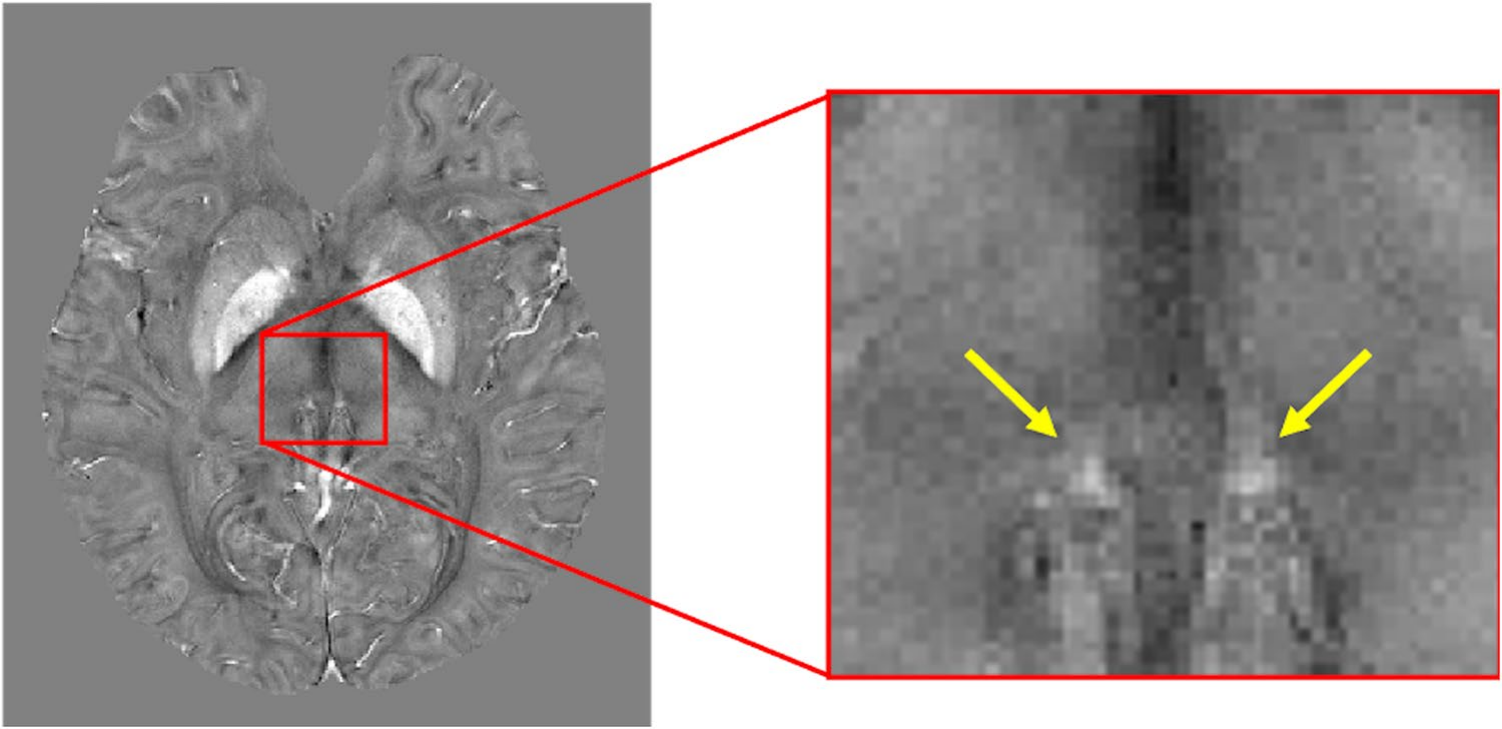
Example of magnetic susceptibility map around the habenula (yellow arrows) on an axial slice.

The magnitude and phase images from the multi-echo GRE scans were combined across the 20 receiver channels by an in-house Matlab code; the code added complex images from different channels after subtracting channel-specific receiver phase maps which were obtained by extrapolation of the measured phase to the zero echo time (TE = 0). After coil combination, R2* was calculated by mono-exponential fitting of the Rician noise-corrected magnitudes of the multi-echo GRE data^46,47^. Since the B0 map in the deep brain region containing the habenula was homogeneous, no additional algorithms^39,48^ for B0 inhomogeneity correction were applied. QSM was reconstructed from the channel-combined magnitude and phase data through the publicly available STI Suite pipeline (https://people.eecs.berkeley.edu/~chunlei.liu/software.html). Here, the raw image phase was first unwrapped using a Laplacian-based method, and the background field was removed by the V-SHARP^49^ algorithm with the radius parameter of 25 mm. Finally, the dipolar field inversion was achieved by STAR-QSM^40^ for streaking artifact reduction. The static field direction in the image coordinates was extracted from the image file header and explicitly taken into account in the inversion process. Figure 4 shows an example of the reconstructed susceptibility map on an axial slice containing the habenula region.

#### Segmentation and adjustment of ROI

**Figure 5:**
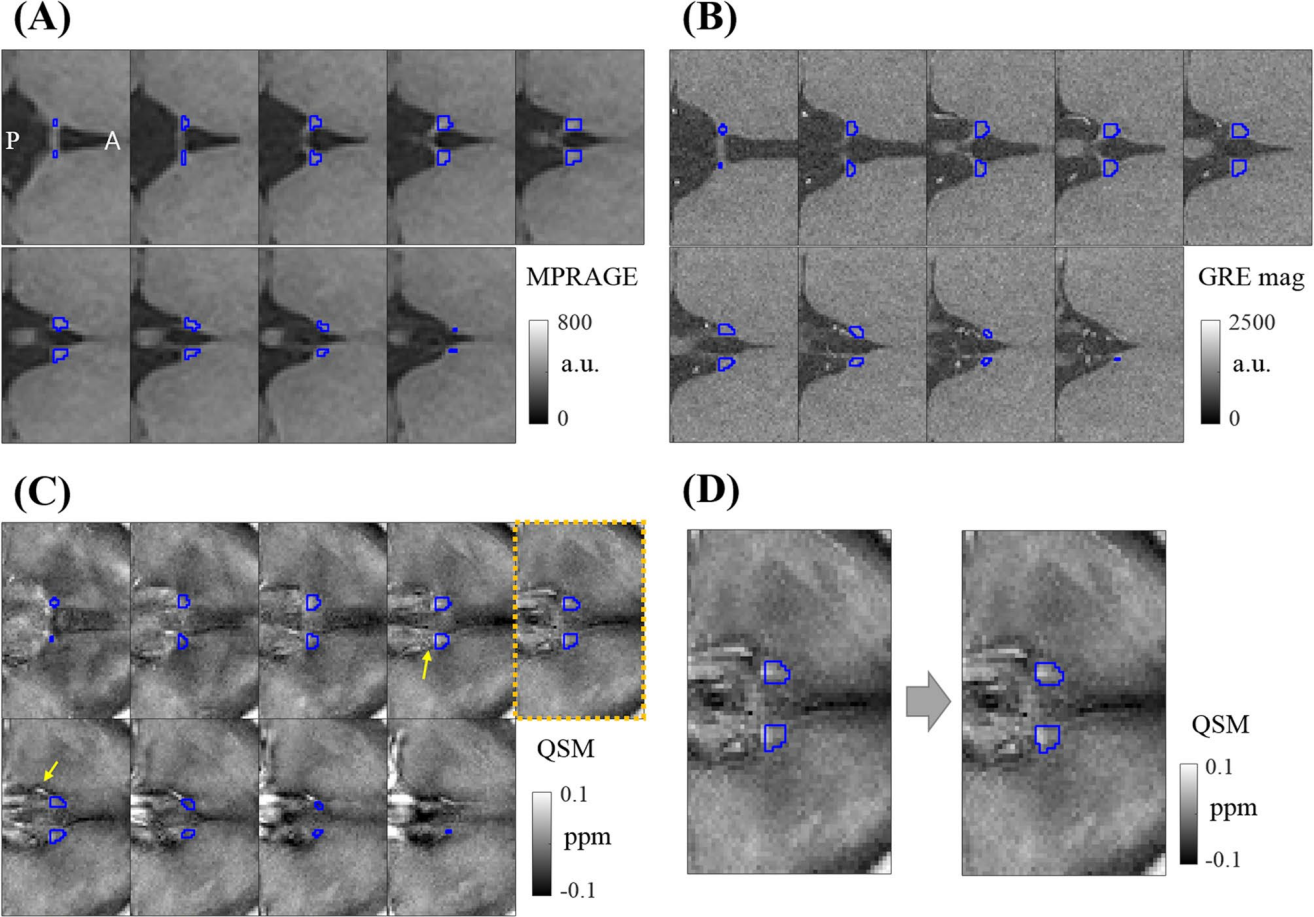
(**A**) Habenula ROI on T1-weighted MPRAGE images following the Lawson’s method. All images are shown on the axial plane, with the anterior side to the right. (**B**) Habenula ROI registered onto GRE magnitude images. (**C**) Same ROI on QSM. Suspected vein-like structures are marked with yellow arrows. The highlighted slice (rightmost) indicates the slice chosen for further analysis (minimum-vein slice). (**D**) Posterior edge adjustment of the habenula ROI on the chosen slice.

We first segmented the bilateral habenula on the coronally reformatted MPRAGE images according to Lawson et al.^26^. The segmented habenula masks were then registered to the GRE images through volumetric, rigid-body image registration of the whole brain T1-weighted image onto the GRE magnitude image by ANTs (Fig. 5A,B). The interpolation threshold parameter was empirically set to 0.45. This yielded the average volume of the habenula (unilateral) of 32.2 mm^3^, which was consistent with the previously reported values in the range of 30–36 mm^3^^9^. Note that the QSM, R2*, and the GRE images were all in automatic alignment since QSM and R2* were derived from multi-echo GRE images.

As reported by He et al.,^12^ we found that the veins in and near the habenula presented a serious potential source of error in susceptibility analysis in the habenula. The degree of venous interference varied widely among subjects. In order to minimize signal contamination in both QSM and R2* maps, and to apply a uniform processing pipeline for different subjects, we decided to limit our susceptibility analyses to a single axial slice. This slice, which we call minimum-vein slice, was selected by the following procedure. First, out of all the axial slices that contained the habenula, we checked off slices where venous intrusion in the habenula was suspected. This was assessed by the presence of elongated (typically one or two-pixel wide) bright features in either the QSM or the R2* map. Of the remaining slices, we selected the slice where the ROI appeared to be the farthest removed from suspected vein-like features, while still containing at least 16 pixels. When there were multiple comparable slices, we chose the one with the largest ROI area. This procedure could be followed in all (48) scan datasets. A representative minimum-vein slice is shown in Fig. 5C,D.

After thus reducing our main analysis volume to a single slice, we performed final manual adjustment of the (now 2D) habenula ROI to better conform to the posterior edge of the habenula trigone on the axial GRE magnitude images (first echo, Fig. 5D)^12^. This was necessary because the initial, coronal-plane segmentation sometimes missed voxels on the habenula’s posterior edge, which was important given the prevalence of susceptibility elevation in the posterior subregion of the habenula.

**Figure 6:**
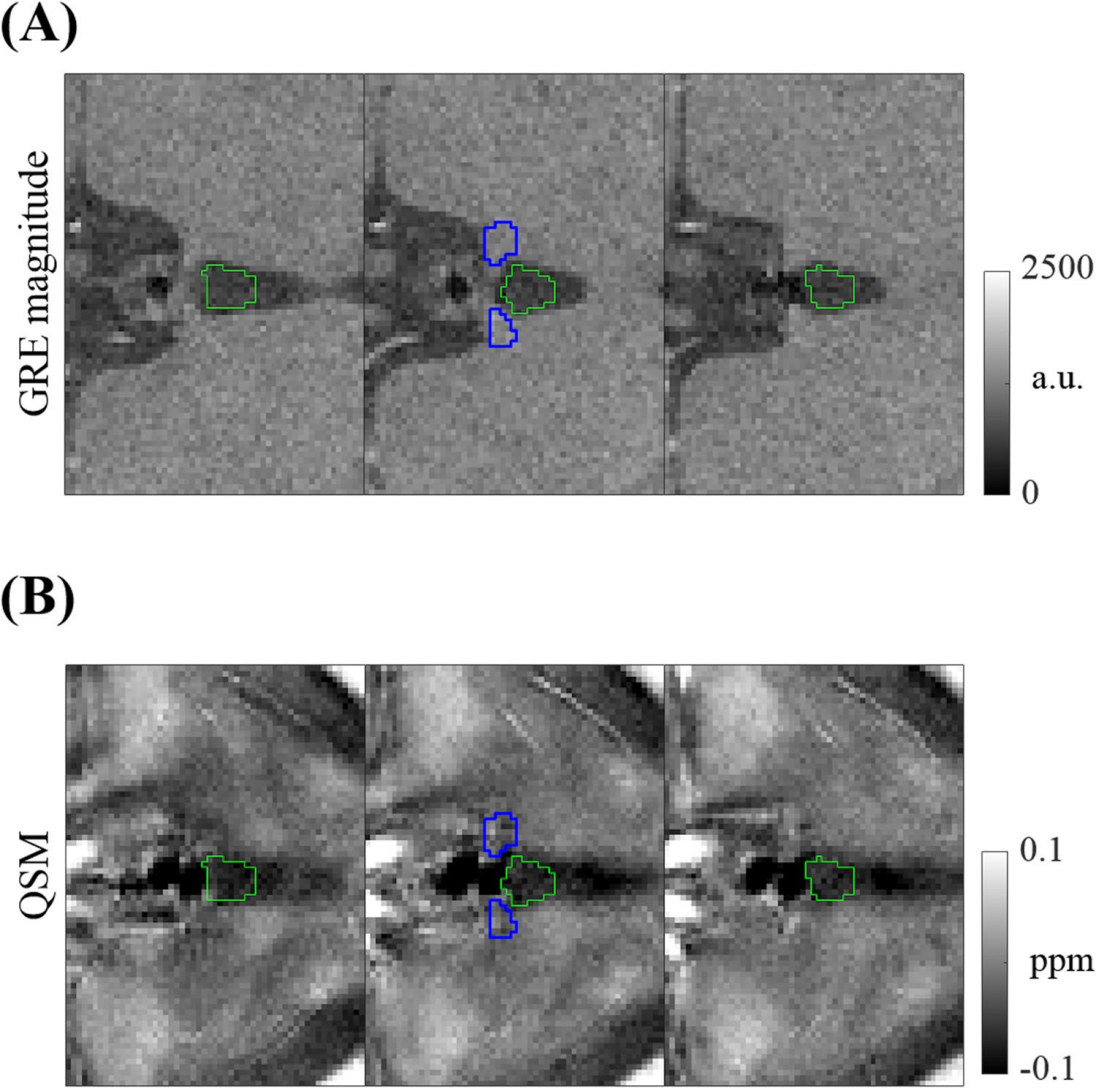
ROI (green) for susceptibility reference in the 3rd ventricle, shown on three consecutive slices of GRE magnitude (**A**) and QSM images (**B**) for a subject with a medium contrast score (volunteer 1, score = 2). Habenula ROI is shown in blue on the middle slice.

The susceptibility reference region was taken from the CSF of the third ventricle. For this, we manually segmented a triangular region in the CSF on three contiguous slices in the GRE first-echo magnitude maps, centered on the minimum-vein slice (Fig. 6). The average of the susceptibility values in the segmented volume served as the susceptibility reference for a given QSM dataset. Note that such reference is not needed for the susceptibility difference and CNR calculations.

#### Visual assessment

**Figure 7:**
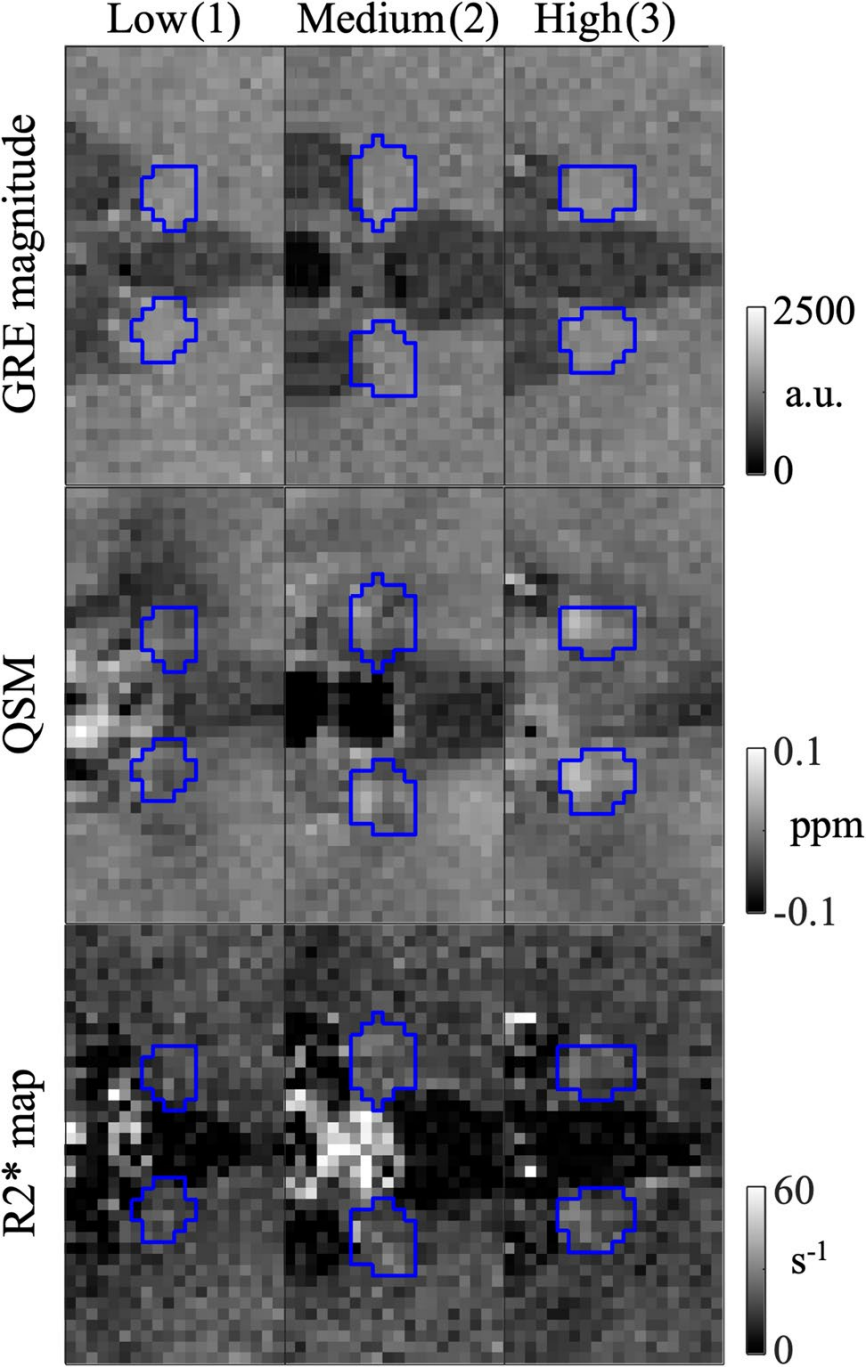
Habenula ROIs (blue outline) on GRE magnitude, QSM and R2* maps of three subjects (volunteer number = 4, 1, 3) from the low, medium, and high contrast score groups. Scores (rater-averaged) are indicated in the parentheses.

The contrast appearance of the habenula in a single-slice axial QSM was visually assessed by three of the authors (JFS, a physician and MR physicist with 40 years of neuroimaging experience; JWK, an MRI scientist; SKL, an MR physicist) according to the scale of low (= 1), medium (= 2), and high (= 3) conspicuity. The three scores were defined as follows. (1) Low: There is no evidence that the habenula as a whole is hyperintense compared to the adjacent thalamus, or there is local hyperintensity within the ROI. (2) Medium: There is some hyperintensity in the whole habenula or locally within the ROI, on at least one of the two sides. (3) High: There is clear hyperintensity in the whole habenula, or locally within the ROI, for both the left and the right sides. Figure 7 shows axial susceptibility maps of representative volunteers in each scoring group (rater-averaged scores of 1 ≤ s < 1.5, 1.5 ≤ s < 2.5, 2.5 ≤ s ≤ 3, along with GRE magnitude and R2* maps on the same slices. The agreement between the three observers was evaluated by the intraclass correlation coefficient (ICC)^50^ calculated in SPSS.

#### Correlation analysis

For each volunteer (and each repeated scan), we plotted the R2* values as a function of the CSF-referenced susceptibility for all the voxels in the single-slice habenula ROI. The Pearson correlation coefficient between the susceptibility and the R2* as well as the linear regression slope were calculated to investigate the sign and the magnitude of the association between the susceptibility and R2*.

#### Contrast analysis

**Figure 8:**
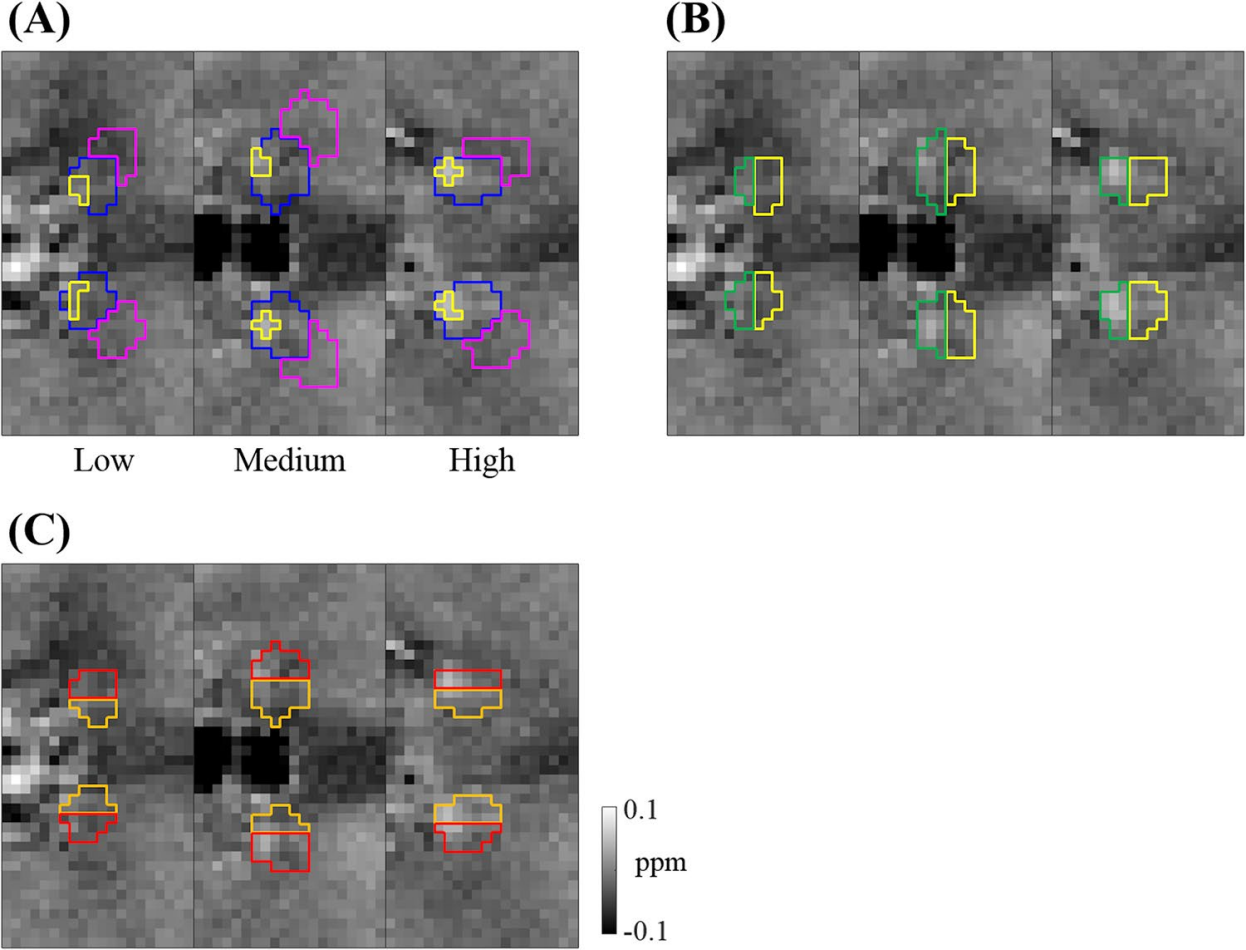
ROIs for CNR calculation displayed on the QSM images of three representative subjects with low, medium, high contrasts (volunteer 4, 1, 3, respectively, from the left to right). (**A**) Peak susceptibility region (yellow) inside the habenula ROI (blue), and the outside region (pink). (**B**) Anterior (yellow) and posterior (green) subregions within the habenula. (**C**) Medial (orange) and lateral (red) subregions within the habenula.

Given the localized nature of susceptibility enhancement in the habenula, we tried several methods to define the susceptibility CNR in the habenula. In all cases, the noise was defined as the standard deviation ${\sigma }_{\chi }$ of the susceptibility in an ROI just outside the habenula in the thalamus. Note that ${\sigma }_{\chi }$ contains contributions from random noise as well as any slow-changing susceptibility variation due, for example, to reconstruction artifacts. This ROI (outer ROI) was obtained by shifting the habenula ROI by half its size both laterally and anteriorly and subtracting the original ROI (Fig. 8A, pink lines). This prescription was found to put the outer ROI in a relatively uniform region of the thalamus with little venous interference. The first CNR calculation method compared the mean susceptibilities in the habenula ROI and the outer ROI.

1 $${\text{CNR}}_{{{\text{mean}}}} { } = { }\left( {\overline{\chi }\left( {{\text{Hb}}} \right) - \overline{\chi }\left( {{\text{outer}}} \right)} \right)/\sigma_{\chi } \left( {{\text{outer}}} \right).$$

This method probed the overall susceptibility enhancement in the habenula. The second method contrasted the peak susceptibility in the habenula (Fig. 8A, yellow lines) with the rest of the habenula. Here the peak value was defined as the average of the five-voxel cluster that contained the maximum susceptibility voxel within the habenula ROI; the cluster was grown by designating the maximum voxel as the seed and then adding the next highest adjacent voxels sequentially.

2 $${\text{CNR}}_{{{\text{peak}}}} { } = { }\left( {\overline{\chi }\left( {{\text{peak}}} \right) - \overline{\chi }\left( {{\text{rest}}} \right)} \right)/\sigma_{\chi } \left( {{\text{outer}}} \right).$$

In the third and fourth methods, the habenula ROI was simply geometrically divided into two halves: anterior/posterior (Fig. 8B) or medial/lateral (Fig. 8C) subregions. These served to investigate the first-order spatial gradient of the susceptibility hyperintensity within the habenula.

3 $${\text{CNR}}_{{{\text{AP}}}} { } = { }\left( {\overline{\chi }\left( {{\text{posterior}}} \right) - \overline{\chi }\left( {{\text{anterior}}} \right)} \right)/\sigma_{\chi } \left( {{\text{outer}}} \right),$$

4 $${\text{CNR}}_{{{\text{ML}}}} { } = { }\left( {\overline{\chi }\left( {{\text{lateral}}} \right) - \overline{\chi }\left( {{\text{medial}}} \right)} \right)/\sigma_{\chi } \left( {{\text{outer}}} \right).$$

#### Grouping of scan data and reproducibility analysis

The processing steps outlined above were applied to all the 48 scan datasets. For three volunteers who were scanned 10 times, we chose one representative scan out of 10 in order to form a group of 21 unique-subject scan datasets. The representative scan was defined as the one which minimized the sum of the normalized distances, in the parameter space, from the 10-scan averages of the following parameters: bilateral habenula volume, mean susceptibility, mean GRE first-echo magnitude, and the mean R2* (mean values were taken in the minimum-vein slice habenula ROI). The quantitative parameters for the habenula, including the size, susceptibility-R2* correlation, and the CNR values were separately analyzed for the unique-subject data group (21 scans) and the reproducibility data group (3 volunteers, 10 scans each).

## Supplementary information

Supplementary Information.

## Data Availability

DICOM files (up to 5 GB) of the multi-echo GRE images and the T1-weighted images, as well as Matlab-processed QSM and R2* maps of representative anonymized volunteers, will be uploaded for public access in the digital data repository zenodo.org (10.5281/zenodo.3732418). The main processing codes in Matlab will also be made available from the same repository. Additional data and codes will be provided upon request to the corresponding author.
