# Supplementary material for: Magnetic susceptibility imaging of human habenula at 3 T
Source: Sci Rep. 2020 Nov 9;10:19357. doi: 10.1038/s41598-020-75733-y (PMC7652825; doi:10.1038/s41598-020-75733-y)
Supplement: Supplementary file 1 — Supplementary Information. [file 41598_2020_75733_MOESM1_ESM.pdf]

Supplementary information

for

Magnetic susceptibility imaging of human habenula at 3T

Seulki Yoo, Joo-won Kim, John F. Schenck, Seung-Kyun Lee

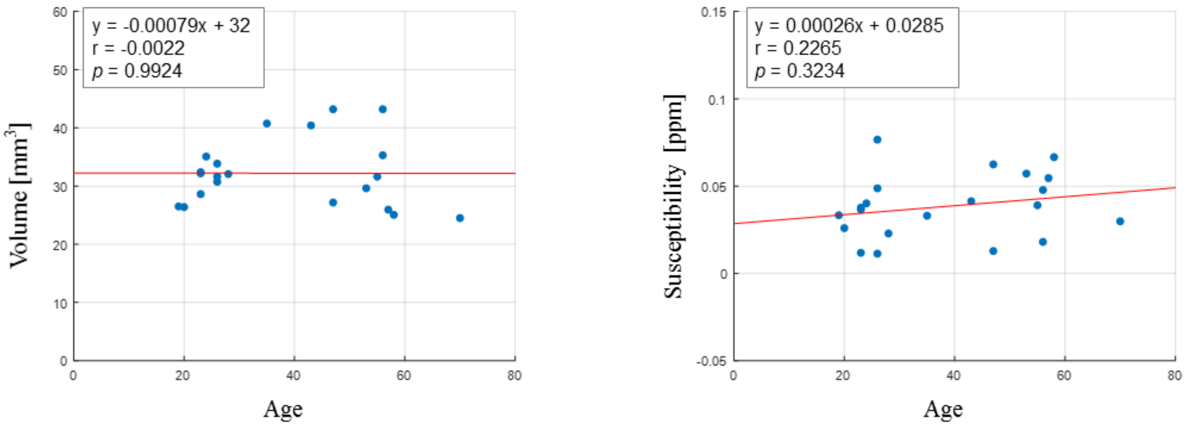

Supplementary Figure 1. Plots of the habenula volume (A) and the mean magnetic susceptibility (B) as a function of the age for the 21 subjects. Solid red lines are linear fits to the data. No significant correlation was found between volume/magnetic susceptibility and age. *r,* Pearson correlation coefficient.

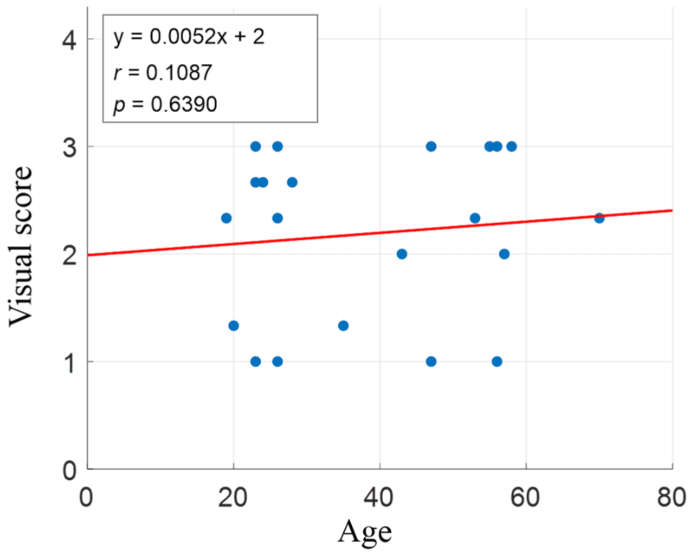

Supplementary Figure 2. Plot of the visual score as a function of the age for the 21 subjects. Solid red line is a linear fit to the data. No significant correlation between visual score and age was observed. *r,* Pearson correlation coefficient.

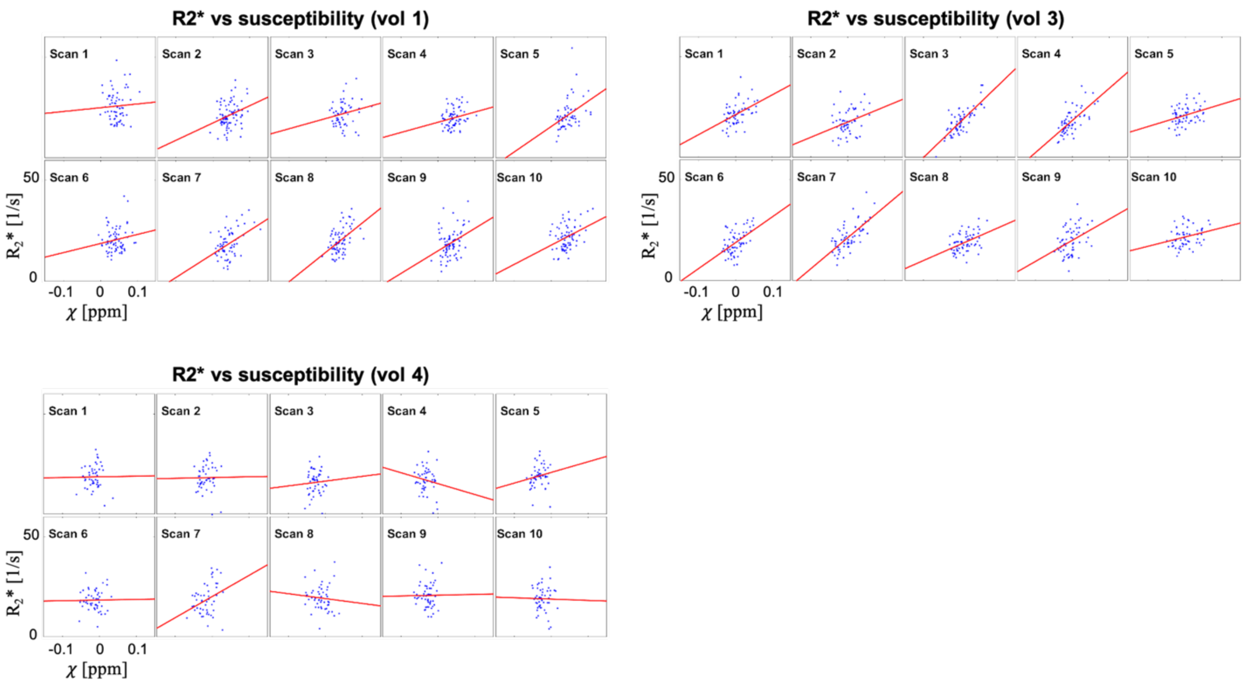

Supplementary Figure 3. R2* vs susceptibility plots for the three subjects with 10 repetitions. Solid red lines are linear fits to the data.

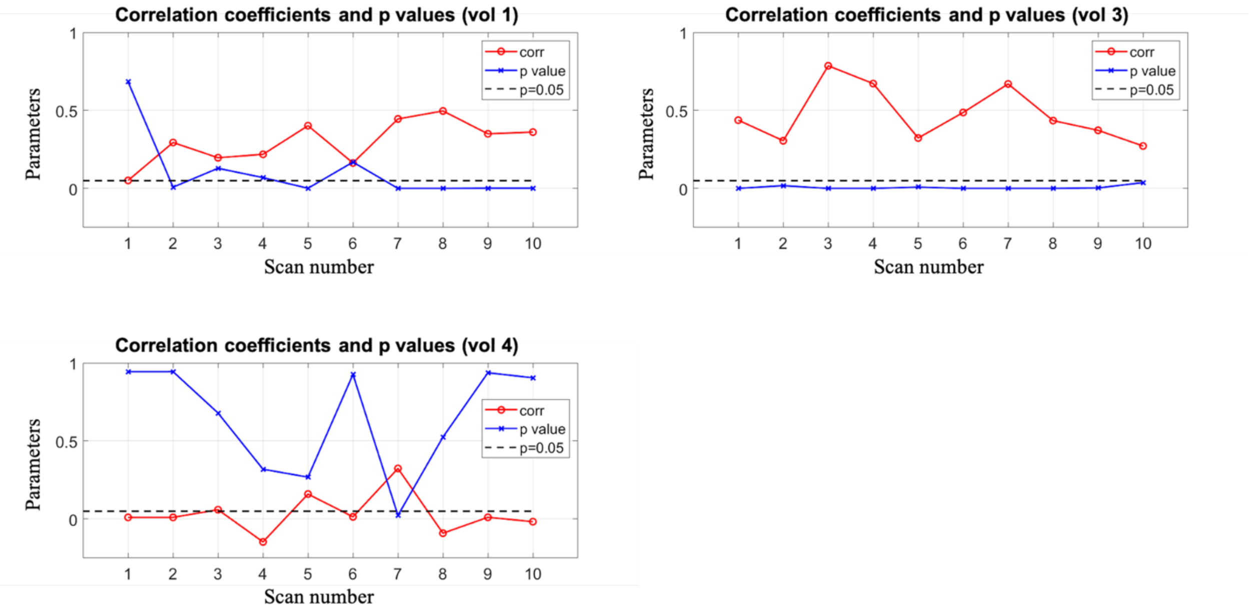

Supplementary Figure 4. Pearson correlation coefficients (red) between susceptibility and R2* in the habenula and the associated *p* values (blue), for the three subjects with 10 repetitions. The black dashed line indicates a *p* value of 0.05.

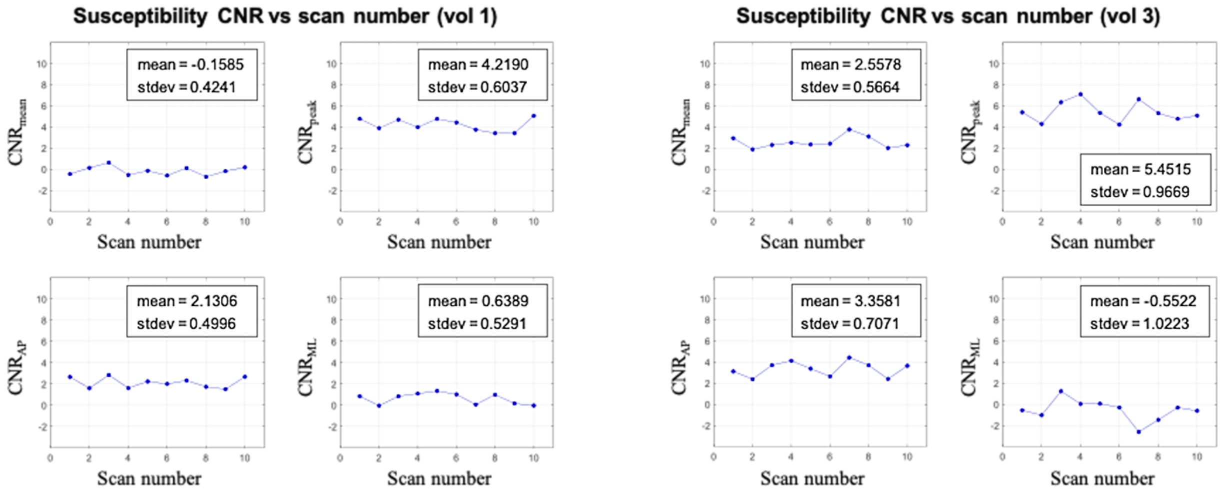

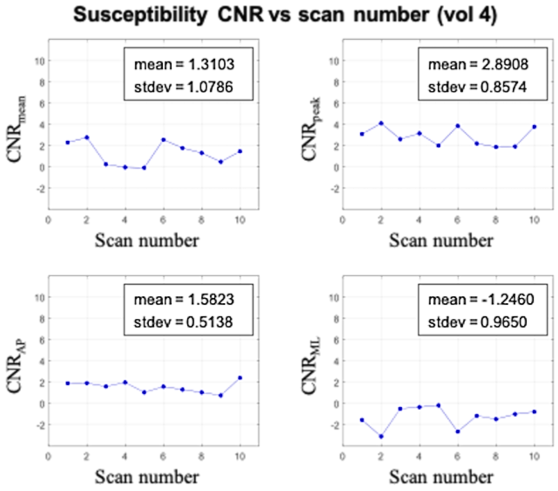

	CNR_{mean}	CNR_{peak}	CNR_{AP}	CNR_{ML}
vol 1	0.4241	0.6037	0.4996	0.5291
vol 3	0.5664	0.9669	0.7071	1.0223
vol 4	1.0786	0.8574	0.5138	0.965
avg	0.6897	0.8093	0.5735	0.8388

Supplementary Figure 5. Susceptibility CNR vs the scan numbers for the three subjects with 10 repetitions. The table lists the standard deviation over the repetitions.

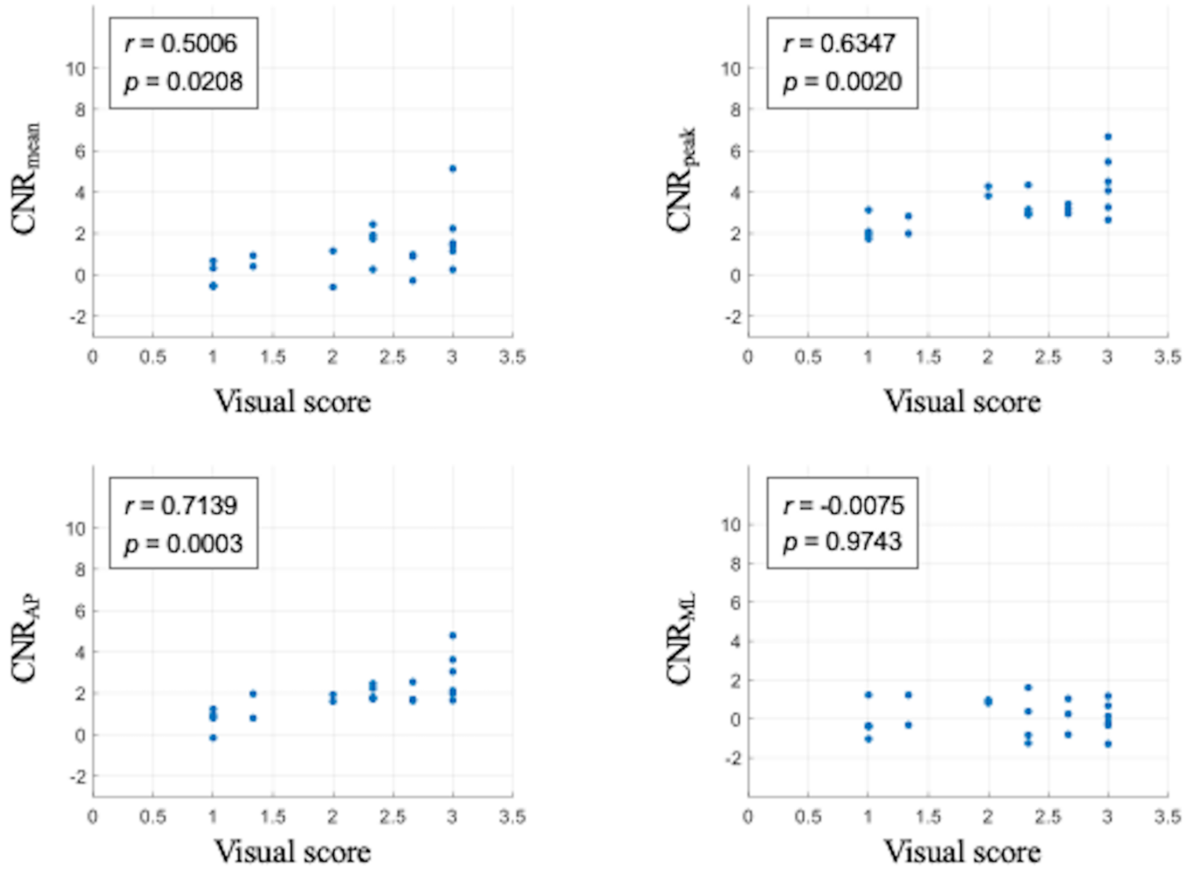

Supplementary Figure 6. Plots of different susceptibility CNRs (with CSF region as noise ROI) vs visual contrast scores for the 21 subjects. The correlation coefficients (*r*) and the *p* values are indicated on each plot.
